# Changes in subfoveal choroidal thickness in myopic children with 0.01% atropine, orthokeratology, or their combination

**DOI:** 10.1007/s10792-021-01855-5

**Published:** 2021-05-05

**Authors:** Qian Hao, Qi Zhao

**Affiliations:** Department of Ophthalmology, The Second Hospital of Dalian Medical University, 467 Zhongshan Road, Shahekou District, Dalian, China

**Keywords:** 0.01% Atropine, Orthokeratology, Myopia, Axial length, Subfoveal choroidal thickness

## Abstract

**Purpose:**

To compare the changes in subfoveal choroidal thickness (SFChT) in myopic children treated with 0.01% atropine, orthokeratology (OK), or their combination in myopic children, and to study the connection between increase in SFChT and axial length (AL) elongation.

**Methods:**

This is a prospective, randomized, controlled study. A total of 67 children were included; 22 patients were randomly assigned to the SA group (patients with spectacles and 0.01% atropine), 24 patients were randomly assigned to the OK group (OK), and 21 patients were randomly assigned to the OKA group (OK and 0.01% atropine). Comprehensive ophthalmologic examinations were performed at baseline, 1 month, 6 months, and 12 months.

**Results:**

After 1 month, SFChT increased by 5.41 ± 1.65 μm in the SA group, 17.46 ± 2.79 μm in the OK group, and 20.19 ± 2.18 μm in the OKA group (*P* = 0.00), whereas AL was not significantly increased. After 12 months, the changes of SFChT were not increased significantly compared with that at 1 month; AL increased by 0.20 ± 0.03 mm in the SA group, 0.28 ± 0.03 mm in the OK group, and 0.14 ± 0.03 mm in the OKA group (*P* = 0.00). The change in SFChT at 12 month was negatively correlated with the change in AL at 12 months.

**Conclusion:**

The control of AL elongation was better in SA group than OK group. The increase in SFChT was best in OKA group, followed by OK group, and the changes were significant after only 1 month. In addition, the increase in SFChT may influence AL elongation and myopia progression.

## Introduction

The prevalence of myopia and high myopia has attracted worldwide attention, especially in East Asia [1]. Myopia is the most common refractive error, and is influenced by various factors, including environment, lifestyle, education, and heredity [2, 3]. Childhood-onset myopia is associated with the development of high myopia, which can result in several pathological complications such as macular degeneration, posterior sclera staphyloma, retinal detachment, and even blindness [4]; the visual impairment is usually irreversible. Therefore, it is critical to implement measures to control the development of myopia and to explore the underlying mechanisms of myopia.

The mechanisms of myopia are still not clear; a potential biological basis is eye growth. Many experiments with animals, including chicks [5, 6], macaque monkeys [7], and marmosets [8], have suggested that imposed defocus on the retina can result in an increase in SFChT and thus influence AL elongation. Similar results have been found in humans [9–11]. Changes in SFChT may play an important role in myopia progression.

OK is a very popular optical method to control myopia progression, and the specific mechanism of OK lens reducing the progression of myopia is unknown. Many previous studies have reported that the effects may result from peripheral retina defocus with an increase in high-order aberration through corneal epithelial redistribution [12–14]. Recently, several studies have reported that the use of OK lens can increase SFChT [15–17] and thus influence AL elongation. For example, Chen et al. [15] found increases in SFChT after short-term treatment with OK lenses. Li et al. [16, 17] demonstrated that the OK lens can induce significant increase in SFChT and retard AL elongation. Zhao et al. [18] showed that the combination of OK lenses and atropine resulted in a greater increase in SFChT than monotherapy with atropine.

Atropine is the effective pharmacological agent to retard myopia progression [19–21]. Many studies have demonstrated administration of 0.01% atropine with a low incidence of side effects and rebound during the washout period [20, 21]. A previous animal study demonstrated that atropine appears to block the M1 and M4 receptors in the retina and sclera that restrain axial elongation and reduce myopia progression by affecting the remodeling of sclera and reducing vitreous chamber growth [22]. And Li et al. [23] have shown that atropine mainly causes reduction in AL elongation. Some studies have reported that atropine can also thicken SFChT by hyperopic retina defocus in animals and humans [24–26]. However, no studies have compared the effects of 0.01% atropine, OK lenses, and their combination on long-term changes in SFChT and AL. The purpose of this study was to compare the effects of these three treatment regimens on long-term changes in SFChT and AL and to study the association of changes in SFChT with AL elongation.

## Materials and methods

### Patients

We included 75 children aged 8–12 years with myopia from the Second Affiliated Hospital of Dalian Medical University between June 2019 and September 2020. Before the experiment, we explained the expected benefits and potential risks of using 0.01% atropine and OK lenses to the parents of children and also informed the detailed methods and precautions. This study was reviewed and approved by the Institutional Review Board of the Second Affiliated Hospital of Dalian Medical University (Dalian, China) and adhered to the tenets of the Declaration of Helsinki. In addition, consent was obtained from the parents of patients. Explanation was provided to children using an easy-to-understand method before obtaining informed consent. Eight participants did not successfully complete the study during the follow-up visits: three because of difficulty in complying with the use of 0.01% atropine eyedrops, two because of keratitis, and three because of lost visits.

### Inclusion and exclusion criteria

The inclusion criteria were as follows: (1) cycloplegic spherical equivalent refraction (SE) at least − 1.00 diopters (D) and diopter of spherical within − 1.00 to − 6.00 DS in both eyes; (2) myopic astigmatism ≤ − 1.00 DC and less than or equal to half the spherical diopter; (3) anisometropia of no more than 1.50 D.

The exclusion criteria were as follows: (1) wearing contact lenses within 3 days of the start of examination; (2) children with ocular disorders such as glaucoma, cataract, keratopathy, strabismus, and amblyopia and systemic disorders such as cardiac and respiratory illnesses; (3) intraocular pressure (IOP) of > 21 mmHg and difference between the eyes of > 8 mmHg; (4) use of anticholinergic and cholinergic drugs that affect the evaluation of efficacy, such as atropine, pirenzepine, and pilocarpine, within the past 1 month; (5) use of other therapies that may affect the evaluation of efficacy within the past 3 months, such as wearing OK lenses and therapy of traditional Chinese medicine; (6) low birth weight (≤ 1500 g); and (7) history of hypersensitivity to atropine or anticholinergic drugs.

## Methods

All subjects underwent comprehensive tests before treatment, including slit-lamp examination, visual acuity testing, autorefraction, intraocular pressure, AL, corneal topography and optical coherence tomography (OCT). We applied 1% atropine eye gel 3 times a day for 3 days for cycloplegic autorefraction. Cycloplegic spherical equivalent (SE) ≥   − 1.00 D was considered to indicate myopia. Subjects were prescribed 0.01% atropine eye drops to be applied once per night before bedtime in both eyes in both the SA and OKA groups. Subjects were instructed to wear OK lens on both eyes every night for at least 8 consecutive hours in both the OK and OKA groups. Additionally, OK lenses were worn 10 min after the use of 0.01% atropine in the OKA group. Some measurements, such as SE, AL, and SFChT, were taken after the use of tropicamide to induce sufficient mydriasis to induce the influence of accommodation. Further, 0.01% atropine is produced by Shenyang Xingqi pharmaceutical company (Shenyang, China), and there were no preservatives and the PH value between 3.5 and 4.0 in 0.01% atropine. The OK lens in this study are four-zone reverse-geometry lenses (Euclid Systems Ortho-K; Euclid System Corp., Herndon, USA) with BOSTON EQUALENS II (oprifocona) and a nominal Dk of 127 × 10^–11^  (cm^2^/s) (ml O2/ml_mmHg) (ISO/Fatt). Spectacles were rematched when the SE dropped by 0.5 D and the vision of wearing glasses decreased. OK lenses were rematched when the naked vision during the day reached < 0.5.

In this study, SE was measured using an autorefractor (KR-800, TOPCON, TOKYO, JAPAN). AL was measured using the non-contact Biometer (lenstar LS 900, HAAG-STREIT AG, KOENIZ, SWITZERLAND). SFChT was measured using the optical coherence tomography (CIRRUS HD-OCT, 5000, SINGAPORE). The average of five consecutive measurements by the same technician was used for analysis.

### Statistical analysis

We applied SPSS 26.0 (IBM Corp., Armonk, NY) statistical analysis software for data analysis. All values were described by mean ± standard deviation. The comparisons of baseline age, SE, AL and SFChT and changes in AL and SFChT were used independent sample one-way ANOVA analyses. The Pearson's Chi-square test was used to analyze gender differences among groups. *P* < 0.05 was considered statistically significant. Stepwise multiple linear regression models were used to investigate the association between the change in SFChT at 12 months and the change in AL at 12 months. The standard *β* indicating the relative importance of each variable in comparable standardized units (z scores) was used to evaluate the importance of each variable in predicting weight. A two-tailed *P* value < 0.05 was considered to indicate statistical significance.

## Results

A total of 75 eyes from 75 participants (right eyes) met the above criteria; 67 participants successfully completed the study. No statistically significant differences were found among the three groups in baseline age, gender, SE, AL, and SFChT (Table 1).

**Table 1:** Demographic and biometric and subfoveal choroidal thickness measures (mean ± standard deviation) at baseline of three groups

	SA	OK	OKA	*P* values
*N*	22	24	21	
Age (years)	9.77 ± 1.27	10.13 ± 1.19	10.10 ± 1.22	0.571^a^
M:F	10:12	13:11	11:10	0.826^b^
SE (D)	3.62 ± 0.57	3.66 ± 0.60	4.07 ± 0.74	0.293^a^
AL (mm)	24.91 ± 0.61	25.17 ± 0.52	25.29 ± 0.56	0.097^a^
SFChT (μm)	240.64 ± 19.93	236.83 ± 16.78	235.14 ± 20.33	0.623^a^

*M*: *F* male: female, *SE* spherical equivalent refractive error, *AL* axial length, *SFChT* subfoveal choroidal thickness

^a^Independent sample one-way ANOVA analyses

^b^Pearson's Chi-square test

During follow-up visits, the rate of AL elongation was significantly reduced in the three groups, although the changes were not significant at 1 month. The reduction in AL elongation was greatest in the OKA group (0.14 ± 0.03 mm), followed by the SA group (0.20 ± 0.03 mm). However, the increase in SFChT in the three groups was significant after only 1 month, especially in the OKA group (20.19 ± 2.18 μm) and the OK group (17.46 ± 2.79 μm). There was no significant change in SFChT in the OK group after 12 months (19.33 ± 2.63 μm). The changes in SFChT in the SA group (5.41 ± 1.65 μm at 1 month, 8.09 ± 1.47 μm at 12 months) were also significant, although not enough obvious compared with the OK group and the OKA group (Table 2).

**Table 2:** The changes of AL and SFChT within SA, OK and OKA group

	SA	OK	OKA	*P* values
*AL (mm)*				
Change at 1 m	24.94 ± 0.61	25.19 ± 0.51	25.31 ± 0.56	0.09^a^
Change over 1 m	0.02 ± 0.01	0.02 ± 0.00	0.02 ± 0.00	0.99^a^
Change at 6 m	25.03 ± 0.63	25.31 ± 0.54	25.38 ± 0.58	0.11^a^
Change over 6 m	0.11 ± 0.02	0.14 ± 0.03	0.09 ± 0.02	0.00^a^
Change at 12 m	25.12 ± 0.63	25.41 ± 0.59	25.43 ± 0.59	0.17^a^ 0.17^a^
Change over 12 m	0.20 ± 0.03	0.28 ± 0.03	0.14 ± 0.03	0.00^a^
*SFChT (μm)*				
Change at 1 m	246.00 ± 21.30	254.29 ± 19.24	255.38 ± 22.19	0.27^a^
Change over 1 m	5.41 ± 1.65	17.46 ± 2.79	20.19 ± 2.18	0.00^a^
Change at 6 m	247.32 ± 21.08	255.71 ± 19.01	257.38 ± 22.04	0.23^a^ 0.23^a^
Change over 6 m	6.73 ± 1.42	18.75 ± 2.65	22.19 ± 2.01	0.00^a^
Change at 12 m	248.73 ± 21.06	256.17 ± 19.03	259.29 ± 21.73	0.22^a^
Change over 12 m	8.09 ± 1.47	19.33±2.63	24.14 ± 1.93	0.00^a^

*AL* axial length, *SFChT* subfoveal choroidal thickness

^a^Independent sample one-way ANOVA analyses

**Fig. 1:**
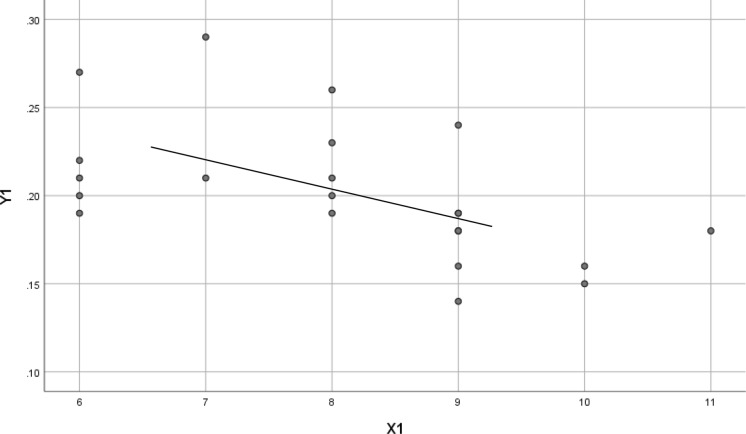
Scatter plots of 12-month change in AL and 12-month change in SFChT. *X*1 = Change in SFChT at 12 months in the SA group. *Y*1 = Change in AL at 12 months in the SA group. Standard *β*,− 0.544; *t* =  − 2.903; *P* = 0.009

**Fig. 2:**
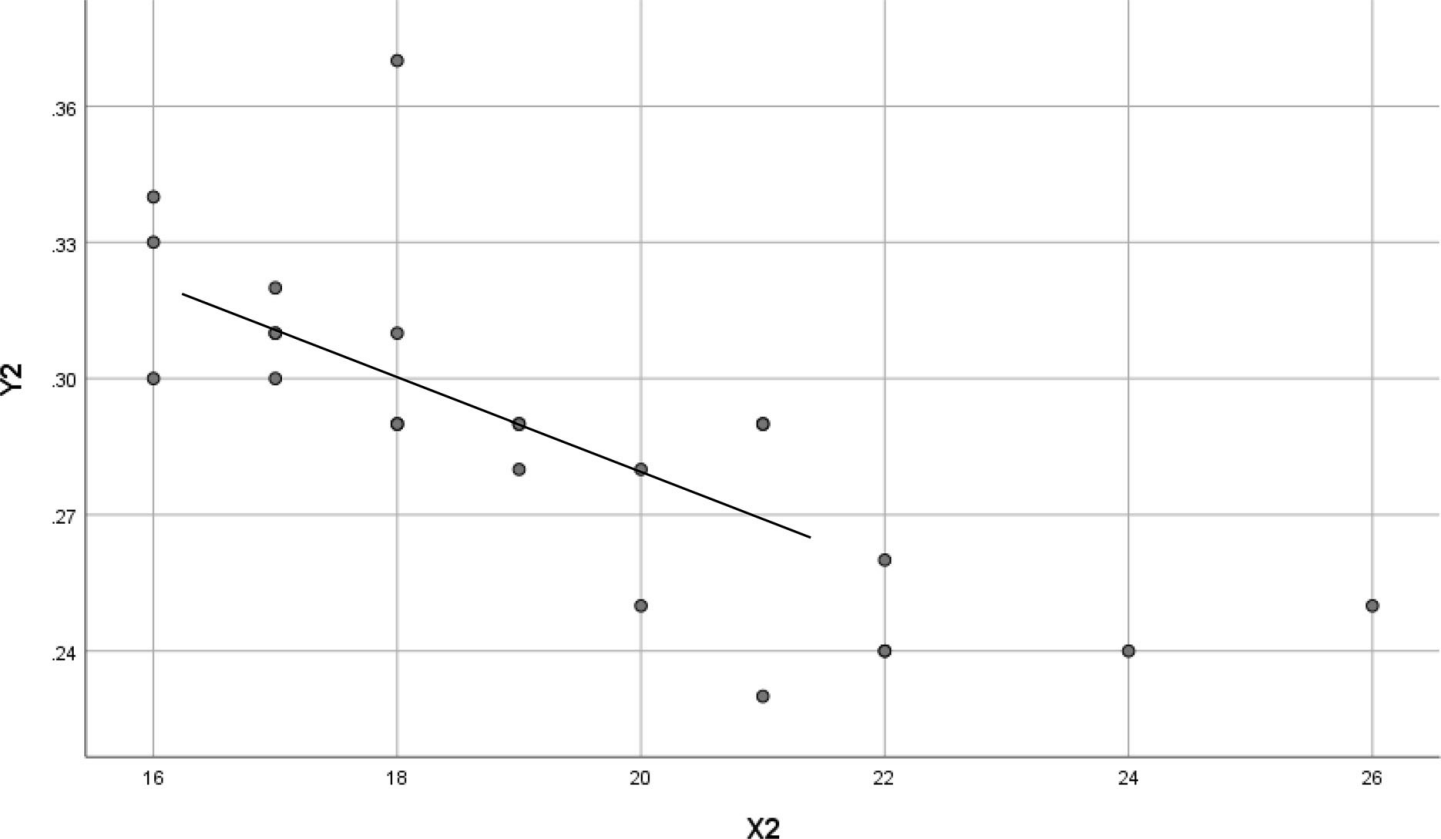
Scatter plots of 12-month change in AL and 12-month change in SFChT. *X*2 = Change in SFChT at 12 month in the OK group. *Y*2 = Change in AL at 12 months in the OK group. Standard *β*− 0.771; *t* =  − 5.679; *P* < 0.001

**Fig. 3:**
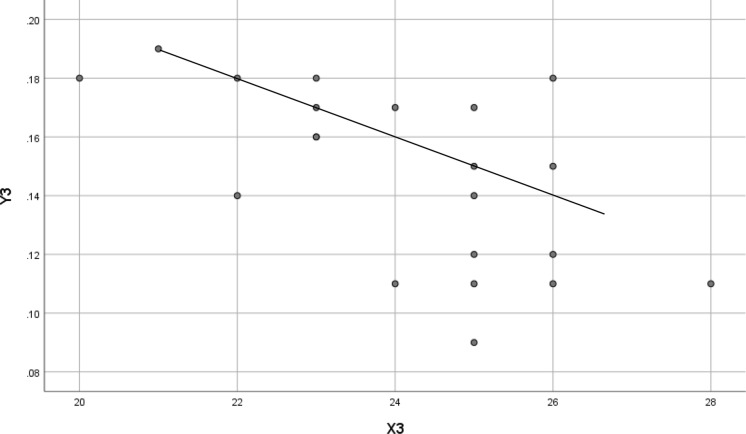
Scatter plots of 12-month change in AL and 12-month change in SFChT. *X*3 = Change in SFChT at 12 month in the OKA group. *Y*3 = Change in AL at 12 months in the OKA group. Standard *β*, − 0.598; *t* =  − 3.255; *P* = 0.004

The scatter plots in Figs. 1, 2 and 3 show that the change in SFChT at 12 months was negatively associated with the change in AL at 12 months in the SA group (standard *β* − 0.544; *t* =  − 2.903; *P* = 0.009), the OK group (standard *β* − 0.771; *t* =  − 5.679; *P* < 0.001), and the OKA group (standard *β*,− 0.598; *t* =  − 3.255; *P* = 0.004) (Figures 1, 2, and 3).

**Fig. 4:**
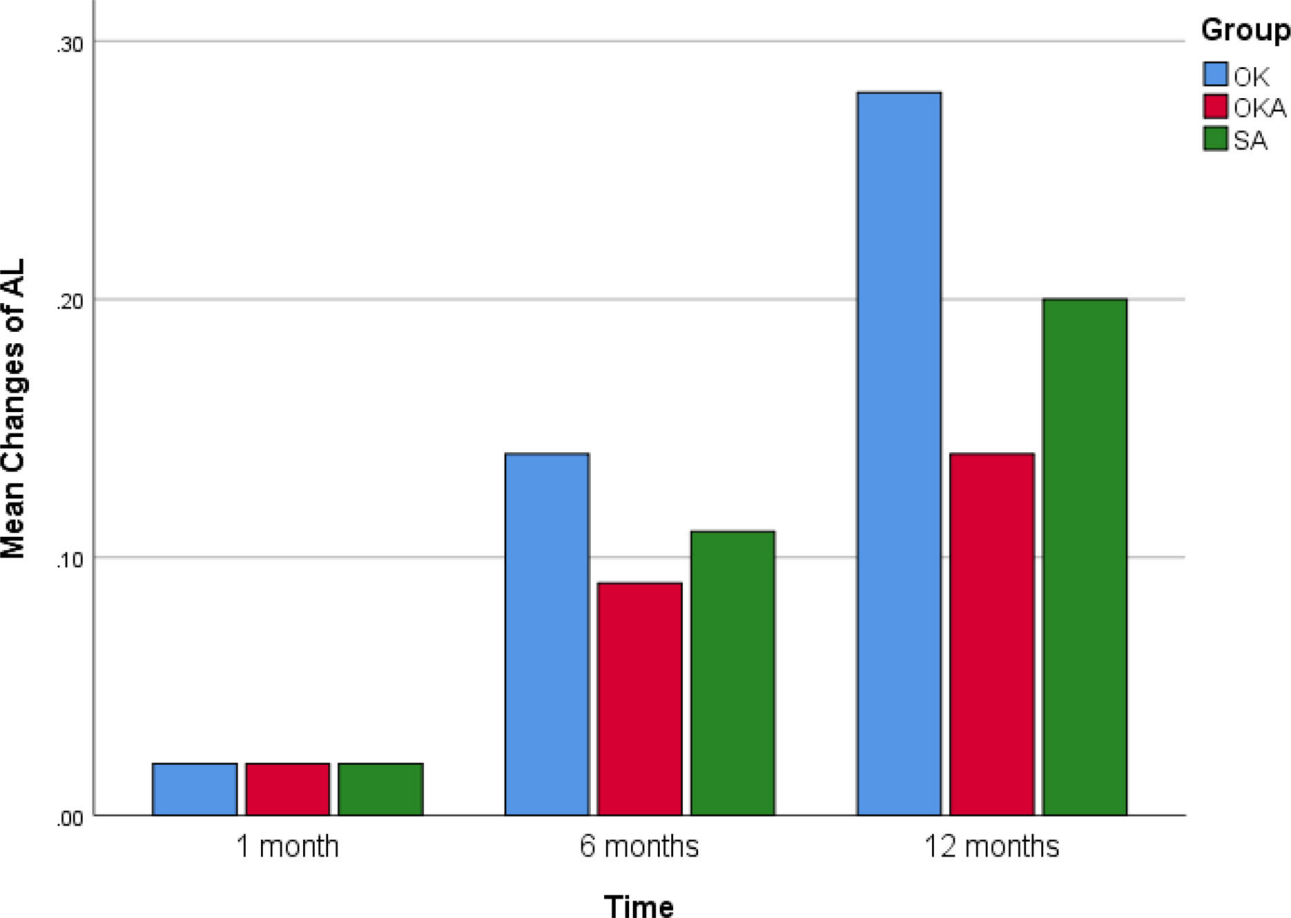
Bar graphic of mean changes of AL

**Fig. 5:**
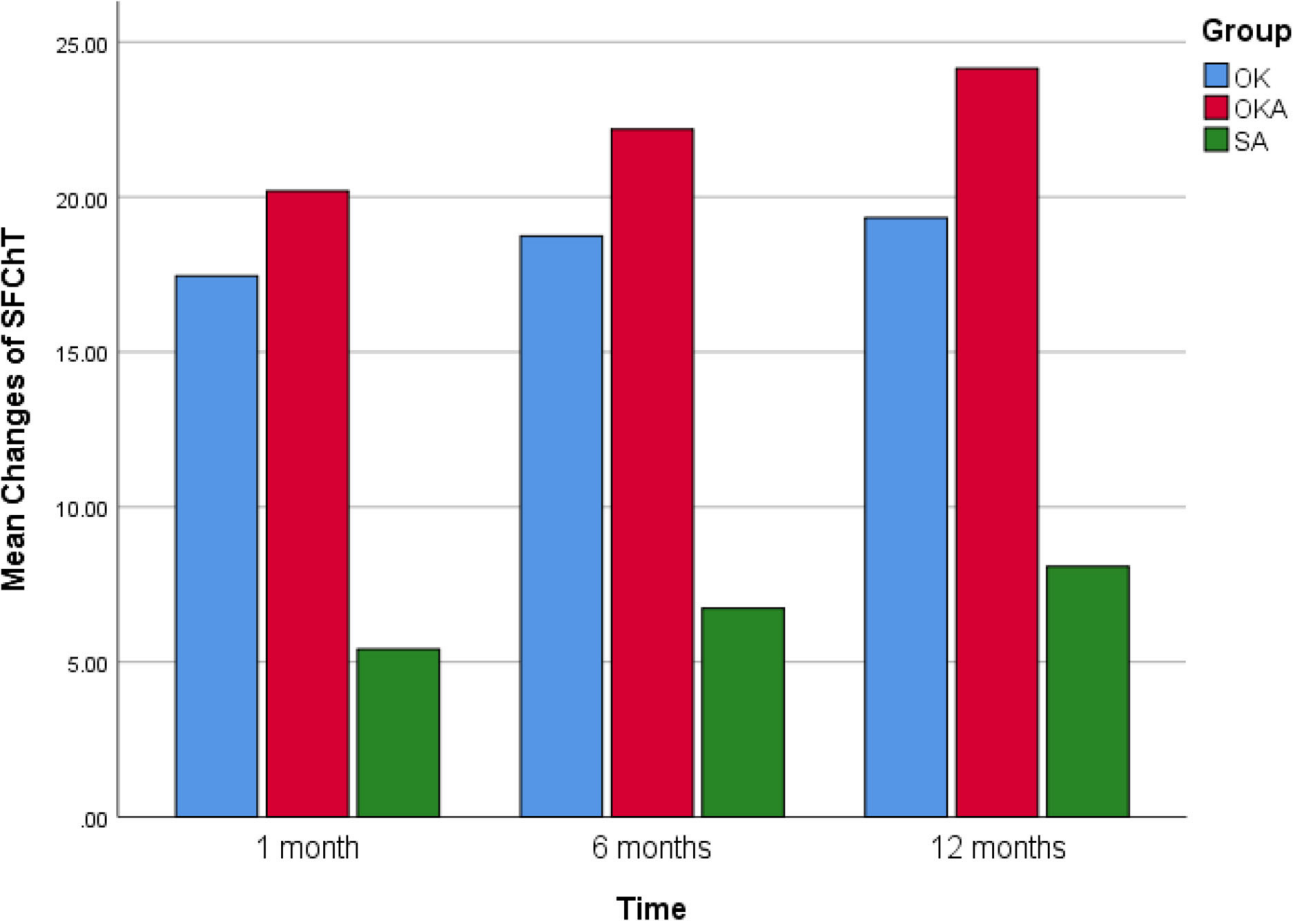
Bar graphic of mean changes of SFChT

The bar graphic of Figs. 4 and 5 show the mean changes of AL and SFChT in OK, OKA and SA at 1 month, 6 months and 12 months (Figures 4 and 5).

## Discussion

Myopia is a refractive error in which the refractive power is too large relative to the axial length of the eye, and uncorrected refractive error is the main cause of visual impairment [1, 27]. Various methods have been used to impede the progression of myopia, including the use of low-concentration atropine [28], pirenzepine, OK lenses [29], peripheral defocus modifying spectacle lenses, rigid gas-permeable contacts lenses, and soft multifocal contact lenses [30]. The uses of 0.01% atropine and OK lens are presently the most common and effective methods.

Previous studies demonstrated that the mechanism of myopia may be related to AL elongation and SFChT thinning. Treatment with 0.01% atropine, OK lenses, and their combination can effectively control the development of myopia and AL elongation, but the specific mechanism needs more research to confirm. Li et al. [23] showed that atropine mainly caused reduction in AL elongation. In this study, we also found that the reduction in AL elongation was greater in the SA group than in the OK group, i.e., the control of AL elongation was better with 0.01% atropine.

Zhang et al. [31] showed that administration of 1% atropine gel for 1 week led to a significant increase in SFChT (20.33 ± 28.41 μm), but this study only measured the effect of a high dose (1%) of atropine. In our study, we found that 0.01% atropine can also increase SFChT; therefore, we speculate that the effect of atropine on the SFChT may be mainly affected by the concentration of atropine. Zhao et al. reported that the increase in SFChT was greater with the combination of OK and atropine than with monotherapy with atropine after 1 month.

However, no previous study has compared the effects of 0.01% atropine, OK lenses, and the combination of them on the increase of SFChT and the change in AL elongation within 1 year. Our study showed that both 0.01% atropine and OK lenses effectively retarded AL elongation and increased SFChT. SFChT was significantly increased after only 1 month in the OK group and remained almost stable, and the increase in SFChT was also significant in the SA group.

The effects on control of AL elongation and increase in SFChT were greatest with the combination of 0.01% atropine and OK lenses; whether there is a superposition effect is unclear. Several studies [32–35] have also demonstrated that the combination of OK lenses and 0.01% atropine is more effective against myopia progression and AL elongation than monotherapy with OK lenses or 0.01% atropine.

This study also showed that the change in SFChT at 12 months was negatively associated with the change in AL at 12 months. Therefore, SFChT may influence AL elongation and myopia progression, but the mechanism is still unknown. We speculate that changes in SFChT may affect the oxygen supply and produce certain chemical substances to provide a sign of slowed axial elongation. Further animal experiments will be required to investigate this hypothesis.

There are also some limitations in this study. First, the change of SFChT may be influenced by the age, SE, accommodation and ethnic of myopia children. Second, our study only used 0.01% atropine, although other concentrations such as 0.5%, 0.1%, 0.05%, and 0.025% are also used clinically. Further studies are required to determine the effect on SFChT with different concentrations of atropine.

## Data Availability

All data and material are available from supplementary material.
